# The Role of Community Mixing Styles in Shaping Epidemic Behaviors in Weighted Networks

**DOI:** 10.1371/journal.pone.0057100

**Published:** 2013-02-20

**Authors:** Yong Min, Xiaogang Jin, Ying Ge, Jie Chang

**Affiliations:** 1 College of Computer Science and Technology, Zhejiang University of Technology, Hangzhou, China; 2 College of Life Sciences, Zhejiang University, Hangzhou, China; 3 College of Computer Science, Zhejiang University, Hangzhou, China; INSERM & Universite Pierre et Marie Curie, France

## Abstract

The dynamics of infectious diseases that are spread through direct contact have been proven to depend on the strength of community structure or modularity within the underlying network. It has been recently shown that weighted networks with similar modularity values may exhibit different mixing styles regarding the number of connections among communities and their respective weights. However, the effect of mixing style on epidemic behavior was still unclear. In this paper, we simulate the spread of disease within networks with different mixing styles: a dense-weak style (i.e., many edges among the communities with small weights) and a sparse-strong style (i.e., a few edges among the communities with large weights). Simulation results show that, with the same modularity: 1) the mixing style significantly influences the epidemic size, speed, pattern and immunization strategy; 2) the increase of the number of communities amplifies the effect of the mixing style; 3) when the mixing style changes from sparse-strong to dense-weak, there is a ‘saturation point’, after which the epidemic size and pattern become stable. We also provide a mean-field solution of the epidemic threshold and size on weighted community networks with arbitrary external and internal degree distribution. The solution explains the effect of the second moment of the degree distribution, and a symmetric effect of internal and external connections (incl. degree distribution and weight). Our study has both potential significance for designing more accurate metrics for the community structure and exploring diffusion dynamics on metapopulation networks.

## Introduction

Structural features (e.g. heterogeneous degree distribution, small-world property, clustering effect, etc.) are critical in explaining epidemic dynamics on complex networks [1]–[8]. Community structure (i.e. the groups of nodes with strong connectivity among their members and weak connectivity to outside nodes) is one of the most important structural features, and broadly exists in social networks, food webs, worldwide trade networks, etc. [9]–[12]. The existence of community structure can restrict the outbreak within a finite number of nodes in the network in the thermodynamic limit [13]. Additionally, the strength of community structure (i.e. the difference between the density of in-community connections and the expected value of that density in a random graph, and as measured by modularity [9]) can strongly affect epidemic dynamics [14]. Moreover, epidemic on metapopulation networks is also closely related to community structures [2], [15]. Recently, studies demonstrated that a weighted network shows different mixing styles, i.e. the pair of connection density and weight distributions among communities [16], [17]. For example, some social networks exhibit that only a few nodes can connect to other communities with large-weight external edges [12], but most of the nodes within trade networks have small-weight external edges connecting to other communities [18]. Although the effect of mixing style on epidemic dynamics was implicitly observed in previous studies [16], the impact of various mixing styles was not explored systematically. Here, we try to isolate the effect of the mixing style from other structural features and describe how mixing style affects epidemic dynamics and the immunization strategies in weighted community networks.

In this study, we first develop a random weighted community network model, which generates networks with different mixing styles under the same modularity. Second, we simulate the epidemic dynamics within the generated networks using the standard Susceptible-Infected-Recovered (SIR) model [3], and compare average epidemic size, speed and pattern arising from different mixing styles. Third, we analyze the performance of a targeted immunization strategy with different mixing styles. Finally, we use branching processes to provide a mean-field solution of the epidemic threshold and size of weighted community networks with arbitrary external and internal degree distribution.

## Materials and Methods

### 2.1 The epidemic model

To investigate the spread of an infectious disease within a network, we use a common approach: nodes in a network represent individuals in a population, and the edges between the nodes represent the contacts along which an infection can spread [3], [19]. The network is abstracted by undirected, weighted edges (i.e. all contacts are reciprocal, and the weight of an edge indicates a transmission rate between the two nodes of the edge). Edges always link two distinct nodes (i.e. no self-loops), and there must be only one edge between any single pair of nodes (i.e., no parallel edges). According to the standard SIR model [3], [19], each node has three possible states: (S) usceptible, (I) nfected, or (R) ecovered. Initially, all nodes are susceptible except the one infected source. Then, during a number of discrete time steps, the initial infection can spread through the network, and the simulation is halted once there are no further infected individuals. At each time step, an infected node *i* infects a susceptible neighbor node *j* with a transmission rate *β_ij_*, which is indicated by the weight of edge (*i*,*j*). Infected nodes are toggled from infected to recovered with a recovery rate, *γ*, i.e. the probability of recovery of an infected node per time step.

Based on the SIR model and the structure of the community network, epidemic entropy [5] is introduced to discriminate the role of mixing styles on the spatiotemporal pattern of the epidemic process. Here, the spatiotemporal pattern indicates the distribution of all infected nodes in different communities at a time and the change of the distribution along with the epidemic process. Entropy is a quantity customarily used in information theory to quantify the level of disorder of a signal or system [5]. At each time step, a snapshot of the epidemic pattern is provided by the set of values of the prevalence, *u_x_* = *I_x_*/*M_x_*, in each community *x*, where *I_x_* is the number of infected nodes in the community and *M_x_* is the total number of nodes in the community *x*. We define the normalized entropy function H:
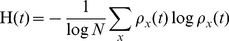
(1)where *ρ_x_* is the normalized *u_x_*, *ρ_x_* = *u_x_*/∑*u_x_*, and *N* is the number of communities in the network. If the epidemic is homogeneously affecting all communities (i.e., all prevalence are equal), the entropy attains its maximum value, H = 1. Starting from H(0) = 0, which represents one initial community with an infected node, H(*t*) increases as more communities becoming infected, thus reducing the level of epidemic heterogeneity.

After a simulation, we calculate the total number of infected cases (the epidemic size), the ratio of the total number of infected nodes to epidemic duration (epidemic speed) and the epidemic pattern over time (epidemic entropy). We use these parameters to describe the epidemic dynamics.

### 2.2 The network model

To generate weighted community networks with different mixing styles under the same modularity, we initialize a network by creating *N* communities consisting of *M* nodes. Each community is constructed using the configuration network model [20] with an arbitrary internal degree distribution *p*(*k*), by which each node in a community is given the appropriate number of *k_i_* of “stubs”, i.e. ends of edges emerging from the node. Pairs of these stubs are then chosen at random and connected to form edges (internal edges). The paring of stubs continues until none are left. The number of stubs must be even, otherwise we simply set *k_i_* = *k_i_*+1 for a random chosen node *i*. A node, therefore, has an average of *d_I_* = ∑*kp*(*k*) internal edges linking to nodes within the same community. Then, we add another *k′* edges to each node with a distribution *q*(*k′*) using the configuration model, making sure that the edges fell only between communities (external edges). Each community has M*d_E_ (*d_E_* = ∑*k′q*(*k′*)) edges connecting to other communities. Thus we create a network with N*M nodes and *E* = 0.5*N*M*(*d_I_*+*d_E_*) undirected edges. Each edge from node *i* to *j* has a weight to represent the transmission rate (0<*β_ij_*≤1) from an infected node to a susceptible one through the edge. In order to maintain the simplest model, the weights of external and internal edges are set equal to *w* and *v* respectively (0<*w*≤*v*≤1). When fixing *N*, *M*, *p*(*k*) and *v*, we adjust *q*(*k′*) and *w* to obtain community networks with different mixing styles among communities but having the same modularity.

The strength of community structure is generally measured as network modularity *Q* [9], [21], which is defined as:
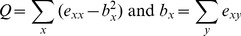
(2)where *e_xy_* is the fraction of all edges in the network that link nodes in community *x* to nodes in community *y*. According to our weighted community network model, in average:
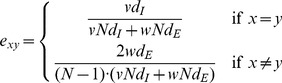
(3)


If edges are to fall between nodes without any regard for the communities, we have a *Q* = 0, and *Q*→1 indicates that networks display strong community structure [9], [21].

**Figure 1:**
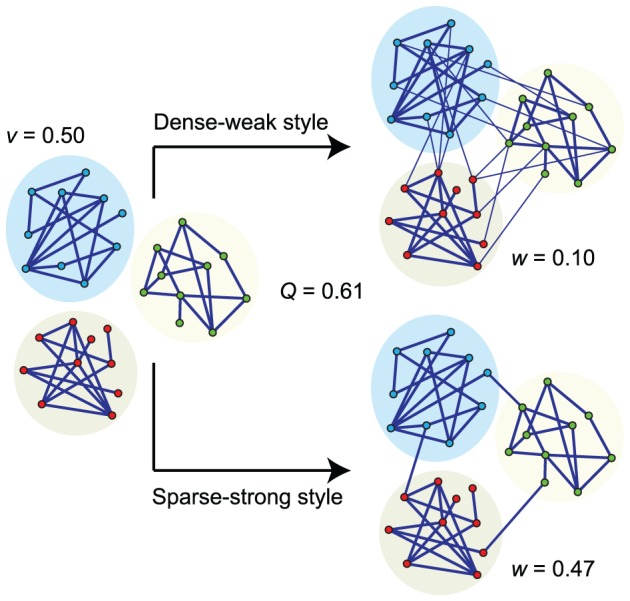
Schematic illustration of sparse-strong and dense-weak styles of weighted community networks. The large circles denote the communities of the network, and the small circles denote the nodes. The lines denote the edges of the network, and its width represents the weight of the edges (i.e., the transmission rate between two nodes). The network parameters are: *N* = 3 (number of communities), *M* = 10 (number of nodes in each community), *d_I_* = 3 (average internal degree), *d_E_* = 0.2 or 1 (average external degree), *v* = 0.5 (weight of internal edges) and *w* = 0.10 or 0.47 (weight of external edges). The modularity *Q* = 0.61 for both styles.

According to the definition of modularity in weighted networks, we define two mixing styles with the same *Q* value. The dense-weak style has a large *d_E_* and a small *w* value. The style has many external edges but these edges has small weights. Contrarily, the sparse-strong style has a small *d_E_* but a large value for *w*. The style is defined by having a few external edges with large weights. In order to compare the two styles, we set *d_E_* = 2/*M* and 1 respectively, unless stated otherwise. A *d_E_* = 2/*M* indicates that only two nodes in each community has an external edge on average, and this is the minimal value to maintain the connectivity of the generated networks. We retain all generated networks consisting of only a single connected component. A *d_E_* = 1 indicates that every node in a community has an external edge on average. An illustration of the difference between two mixing styles with the same *Q* is presented in figure 1.

In order to quantify the difference between two mixing styles, we generate 200 networks for each mixing style, and repeat 10 times of SIR simulation on each generated network with given recovery rate (*γ*) and a randomly chosen initial infected node for each simulation. The average values of three epidemic parameters (epidemic size, speed and entropy) are calculated after 2000 simulations. Based on the comparison of mean values of three epidemic parameters between two styles, the difference from the two mixing styles can be illustrated.

## Results

### 3.1 Simulation results

**Figure 2:**
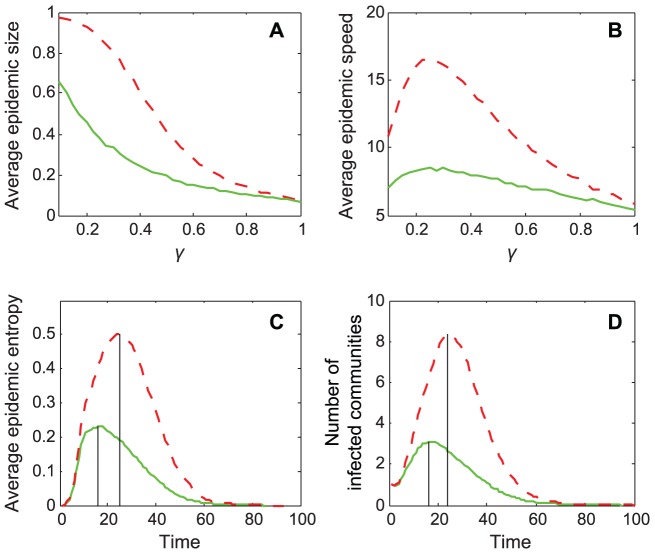
Comparison of Epidemic properties in sparse-strong and dense-weak styles of weighted community networks. (A) average epidemic size (i.e., density of infected nodes) as a function of recovery rate *γ*; (B) average epidemic speed (i.e. the ratio of total infected nodes to total epidemic steps) as a function of recovery rate *γ*; (C) average value of the epidemic entropy as a function of time steps; and (D) average number of infected communities (it has at least one infected node at a time) as a function of time steps. The network parameters are: *N* = 20, *M* = 50, *d_I_* = 4, *d_E_* = 0.04 or 1, *v* = 0.5 and *w* = 0.49 or 0.02. The modularity *Q* = 0.94 for both mixing styles. The recovery rate *γ* = 0.35 in (C) and (D). Solid curves represent sparse-strong style networks and dashed curves represent dense-weak style networks. The vertical lines indicate the appearing time of maximal value of average epidemic entropy and number of infected communities in C and D.

We generate networks with different mixing styles maintaining the same modularity value. We set both the external and internal degree distributions as Poisson distributions, thus the average internal and external degree (*d_E_* and *d_I_*) directly reflect the entire distribution and connection density. According to the simulating results, the average epidemic size and speed are strongly affected by the mixing styles (figure 2A and B). Although these networks have the same number of communities and modularity value, the dense-weak style networks result in a larger epidemic size (maximal 2.8-fold at *γ* = 0.35) and a faster epidemic speed than the sparse-strong style networks (figure 2A and B). The difference of each epidemic parameter between the two styles is obvious, therefore, the mixing style among communities has a significant effect on epidemic size and speed when the modularity values are equal.

In addition to the differences between the final status according to the two styles, epidemic entropy also demonstrates that the spatiotemporal pattern of epidemics depends upon the mixing style. The entropy and number of infected communities over time are shown in figure 2C and D. We find that diseases can rapidly reach more additional communities in dense-weak style networks, and synchronously spread during a long time window. Moreover, the appearance of maximal entropy in the dense-weak style is later than in the sparse-strong style, therefore, the later appearance and higher value of maximal entropy suggest that the end of an epidemic is rapid in the dense-weak style. In summary, the dense-weak style displays a relative “parallel” evolution (H is large) of the epidemics. Contrarily, we observe a different scenario for the sparse-strong style, where the evolution of epidemics is relative “sequential”. The maximum entropy of the sparse-strong style is significantly smaller than that of the dense-weak style, and a relatively slow decrease in the entropy curve indicates a long-lasting spreading effect in a relatively few communities (figure 2C and D). Therefore, the mixing style also affects the spatiotemporal pattern of an epidemic in networks with the same modularity.

**Figure 3:**
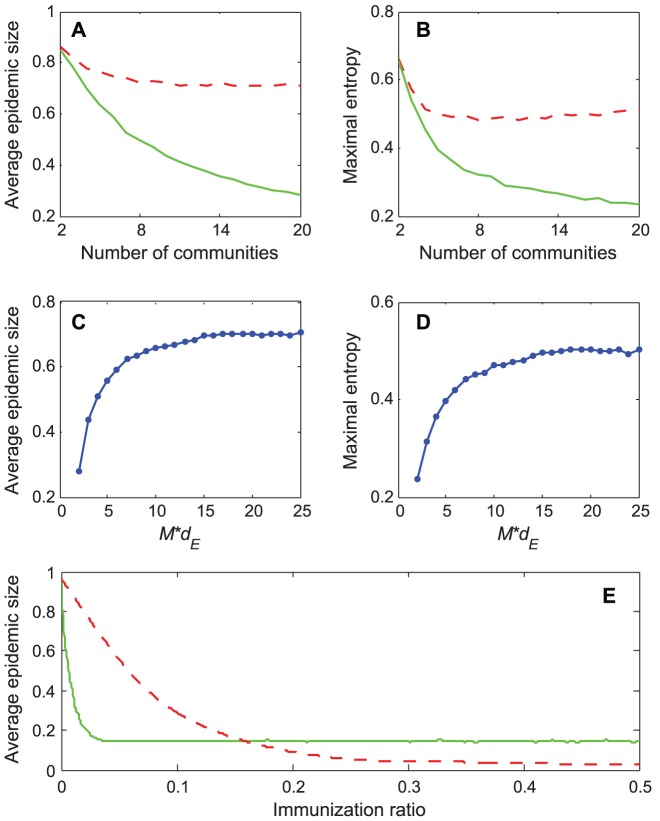
Detail effects of mixing style. (A) and (B) average epidemic size and maximal epidemic entropy as a function of the number of communities. Network parameters are *N* = 2 to 20, *M* = 50, *d_I_* = 4, *d_E_* = 0.04 or 1, *v* = 0.5 and *w* = 0.49 or 0.02. The modularity *Q* = 0.49 to 0.94, and is the same for both styles for each *N*; (C) and (D) average epidemic size and maximal epidemic entropy as a function of the connection density. Network parameters are *N* = 20, *M* = 50, *d_I_* = 4, *d_E_* = 0.04 to 0.5, *v* = 0.5 and *w* = 0.49 or 0.02. The modularity *Q* = 0.94 for all networks with different connection densities; (E) average epidemic size (the ratio of infected nodes to total number of nodes in original networks) as a function of immunization ratio, which is the ratio of nodes with immune state (i.e. cannot be infected). The targeted immunization of nodes is according to the descending order of their external degree. The Network parameters are *N* = 20, *M* = 50, *d_I_* = 4, *d_E_* = 0.04 or 1, *v* = 0.5 and *w* = 0.49 or 0.02. The modularity *Q* = 0.94 for both mixing styles. Solid curves represent sparse-strong style networks and dashed curves represent dense-weak style networks. The recovery rate *γ* = 0.35 in all SIR simulations.

In order to evaluate the effect of the number of communities on the epidemic profiles, we increase the number of communities from *N* = 2 to 20 while keeping all the other parameters the same. We find that the increase in the number of communities actually enhances the distinction between epidemic behaviors on dense-weak and sparse-strong style networks (figure 3A and B). The result shows that each epidemic parameter is obvious different when *N*>4 in the simulation. Although the effect of the mixing style is more obvious in the situation with the inclusion of more communities, the effect cannot be ignored when the number of communities was small.

In order to investigate the effects of the change of external connection density (*d_E_*) in detail, we simulate epidemic spreading for each M*d_E_ (from 2 to 25) with the same modularity, and calculate an average epidemic size and entropy. We find that the epidemic size and maximal entropy change nonlinearly with the increase of *d_E_*, and exhibite a ‘saturation point’ (figure 3C and D). When M*d_E_ is below a point (≈10 in the figure), two parameters change rapidly with the increase of external connection density, such that epidemic size and entropy are more than doubled. When M*d_E_ is above the point, however, the epidemic parameters become relatively stable. This observation is critical because the saturation point of the external connection density can be an important indicator of an epidemic spread within community networks. When the modularity value is the same, the mixing style could be a critical feature in controlling the spread of infectious diseases.

For halting or mitigating an epidemic, targeted immunization interventions or social distancing interventions focusing on changing the structure of the network of susceptible individuals can work in such a way as to make it more difficult for pathogens to spread [22]–[24]. Here, we rank the nodes of a network by their number of external edges as immunization targets. According to our network model setting, the rank reflects both the level of connection and the betweenness of a node in community networks [14], [25], [26]. The sparse-strong style experiences a rapid decrease in epidemic size when a few critical nodes are initially immune (figure 3E), due to the shortage of nodes with external edges in the sparse-strong style. However, the dense-weak style displays a smooth change in epidemic size with the increase of the proportion immunized (figure 3E); because high number of external edges between communities can effectively maintain the connectivity of entire networks. Therefore, the mixing style affects not only the epidemic behaviors on weighted community networks but also the application of immunization strategies.

### 3.2 Mean-field analysis

**Figure 4:**
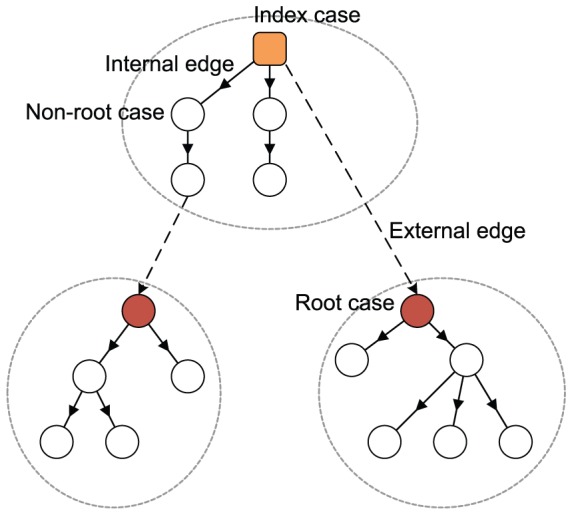
Tree-like structure of infectious components in weighted community networks. Due to the hypothesis regarding the infinite size of the network and community, the infecting processes will not cross the same node or community more than once.

The simulation results described above demonstrate that the mixing style has special effects on epidemic size, speed and pattern, but another important epidemic parameter, global epidemic threshold, is still unclear. Colizza and Vespignani (2007) derived the expression of the epidemic threshold parameter for the case of SIR model in subpopulation networks where communities were coupled via Markovian mobility of individuals [15]. In this section, we use multiple-type branching processes to calculate the mean-field expression of the epidemic threshold and size on uncorrelated networks with arbitrary external and internal degree distribution [27]. According to the network model presented in section 3, we assume that the size of the network and the communities is approximately infinite (*N*, *M*→*∞*) and the recovery rate *γ* = 1 in the theoretical study [20]. In this situation, the epidemic processes form a tree-like infectious component (figure 4): starting from an index case (the initial infected node), the pathogens can infect root nodes through external edges, and infect non-root nodes through internal edges. Here, ‘root’ indicates the node that was the first case infected by other communities. The distribution of potential root and non-root nodes connected to an infected root node can be described by two generating functions:
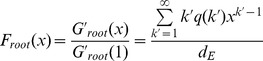
(4)

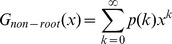
(5)where *F_root_*(*x*) indicates the distribution of the number of external edges of a root node excluding the edge that the pathogen arrived along, *G_root_*(*x*) = ∑*q*(*k*)*x^k^* is the generating function for the external degree distribution and *G_non-root_*(*x*) is the generating function for the internal degree distribution. By considering the different infection probabilities (the weights of edges) of external and internal edges, the distributions of actual numbers of infected cases from a root node follow the functions:
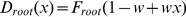
(6)

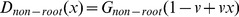
(7)


Similarly, the distributions of the actual numbers of infected cases from a non-root node follow the two generating functions:
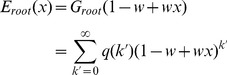
(8)

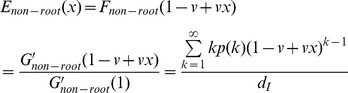
(9)


Therefore, a two-type branching process can describe the approximate tree-like infectious component. To define the behavior of the two-type process, we need two generating functions, each in two variables. The first generating function, *f^root^*(*x*_1_,*x*_2_), determines the distribution of the number of root and non-root nodes to be connected by a root node. Because the external and internal degree distribution is independent, we let:
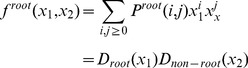
(10)where 0≤*x*_1_,*x*_2_≤1, and *P^root^*(*i*,*j*) is the probability that a root node connects to *i* root nodes and *j* non-root nodes. Similarly, the second generating function, *f^non-root^*(*x*_1_,*x*_2_), determines the distribution of the number of root and non-root nodes to be connected by a non-root node:
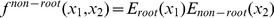
(11)


Let *a_s,t_* = the expected number of type *t* offspring of a single type *s* infected node in one time step (*s*,*t* = root or non-root), hence the mean matrix, *A*, of the two-type branching process is:
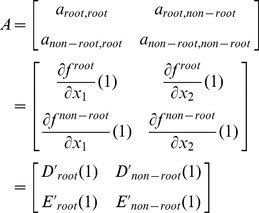
(12)


According to the basic theorem of branching processes, if the matrix *A* has a maximal eigenvalue *eig_max_*(*A*)>1, the extinction probability of the branching process is less than 1, in other words, the pathogen can occupy entire networks. Therefore, the condition of epidemic threshold is:
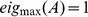
(13)If all *a_s,t_*>0, the condition displayed as:
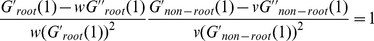
(14)


**Figure 5:**
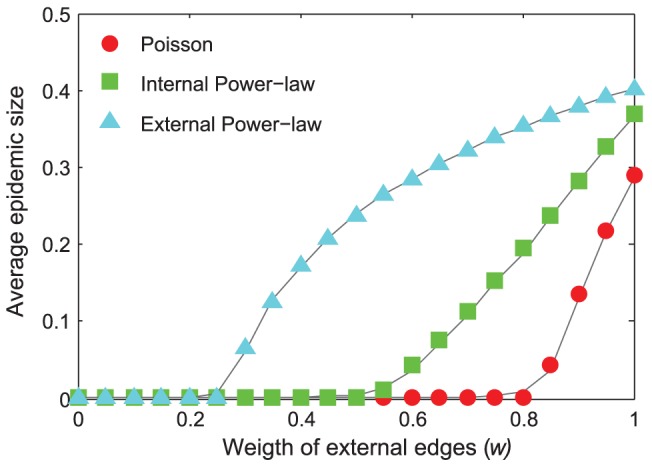
A comparison of the theoretical and the simulated epidemic size and threshold. The curves depict the average epidemic size as a function of the external transmission rates in the simulations, and the points denote the theoretical values of the epidemic size in three situations (calculated using Eq. 16). The Network parameters are *N* = 500, *M* = 500, *v* = 0.2. We use mean value and range to define a degree distribution. The mean value of internal Poisson (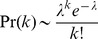) and Power-law distribution (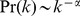) is 1.2, and the mean value of external Poisson and Power-law distribution is 1. The maximal internal degree does not exceed 10 in simulations. There are three combinations of internal and external degree distributions: internal Poisson with external Poisson (red circles), internal Power-law with external Poisson (green rectangles), and internal Poisson with external Power-law (cyan triangles). The recovery rate *γ* = 1 in the SIR simulations.

In addition to epidemic threshold, multiple-type branching process also provides a mathematical solution of epidemic size for our model when *eig_max_*(*A*)>1. If *eig_max_*(*A*)>1, the extinction probability of the two-type branching process represents the probability that a node does not link to giant percolation component in the network. The extinction probability of the two-type process is the smallest nonnegative root *u* = (*u_root_*, *u_non-root_*) of the questions of Eq.10 and 11:
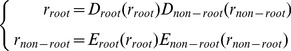
(15)where *r_root_* is the extinction probability starting from a root node, and *r_non-root_* is the extinction probability starting from a non-root node. By randomly choosing a node as index case, the average epidemic size equals to:
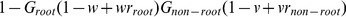
(16)The theoretical threshold and giant component size is identified by the simulation result (figure 5).

The Eq. 14–16 provide us with a clear description of the effect of mixing styles. First, the mean-field analysis extends the mixing style from some certain external and internal degree distribution (e.g. Poisson distribution in ref 13 and power-law distribution in ref 16) to the uncorrelated combination of arbitrary distributions. The equation shows that the second moment of the generating function of the external and internal degree distribution plays a critical role in determining the relation between connection density (*d_I_* and *d_E_*) and transmission rate (*v* and *w*) in the mixing style. Second, the equation demonstrates that the external and internal degree distribution does not simply sum up to determine the epidemic behaviors as the assumption of previous studies in unweighted community networks [13]. Third, the external and internal degree distribution plays a “symmetric” role in affecting epidemic threshold, which complements the previous simulation study [16].

## Discussion

A large number of human infectious diseases spread directly from one person to another, and early works on the spread of such diseases within a network were focused on the heterogeneous distribution of the node degree [3], [14], [19]. When considering additional features of the network structure within real populations, one of the most important findings was the demonstration that the modularity value can strongly affect epidemic dynamics [14]. Our results extend this finding and show that even in weighted networks with the same modularity, fundamentally different epidemic dynamics are observed due to different mixing styles among communities. Second, various mixing styles among communities have a wide existence in real networks [12], [18], and this is the first time that the mixing style is clearly isolated from other structural features to determine nonlinear effects and the epidemic behaviors of weighted community networks. Third, our study supports the idea that data on the strength of community structure will not be sufficient to predict the entire epidemic dynamics related to weighted networks [16]; the mixing style among communities complements the knowledge about community networks.

The impact of mixing style on epidemic behaviors suggests that it should be considered in the detection and measurement of community structure in complex networks. Current measures of community always emphasize the statistical distinction between the connection strengths inside and outside of the communities [11], [21], [28]. However, the effect of mixing style is still ignored in current metrics. Although the degree distribution is one of the most studied statistical features, mixing style is still not considered in the metrics of the community structure. The Eq. 14 clearly shows that external and internal degree distribution has obvious effects on diffusion dynamics in community networks. Therefore, the integration of modularity and mixing style can greatly improve our understanding of diffusion on weighted networks within the community structure.

The effect of mixing style also plays a critical role in the study of epidemic dynamic in networks with metapopulation structures [5], [15], [29]–[32]. A metapopulation consists of a group of spatially separated populations of the same species, which interact at some level. Colizza et al. (2006) showed that the heterogeneous connectivity patterns among subpopulations in airline transportation networks dynamically affected the behavior of the diffusion processes occurring in the networks [5]. Our result regarding the effect of second moments of external or internal degree distributions supports the conclusion. Moreover, multiple-type branching process was used to study epidemic spreading in structured population [27]; our approach extends application of multiple-type branching process to the populations whose structure is described by explicit network structures. In the future, our approach has potential applications in metapopulation networks [5].

In this study, we initially consider the epidemic effect of mixing style among communities. In the future, other aspects of mixing style should also be considered, including the correlation between the internal and external structure of a community, and the dynamic contact structure among communities. Additionally, overlapping community structure is likely to play a critical role in infectious disease dynamics [33], [34], because people are members of multiple, potentially overlapping communities. The exact effect of mixing style among communities on epidemic dynamics is still open for investigation.
